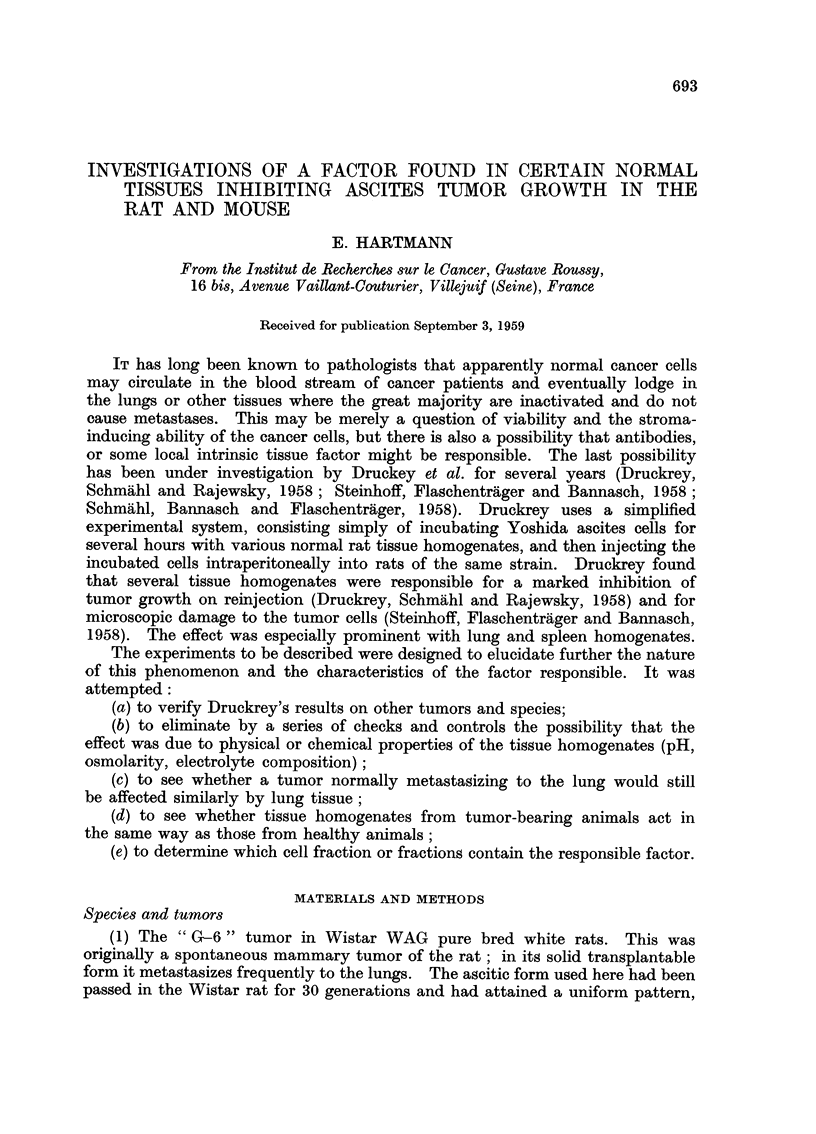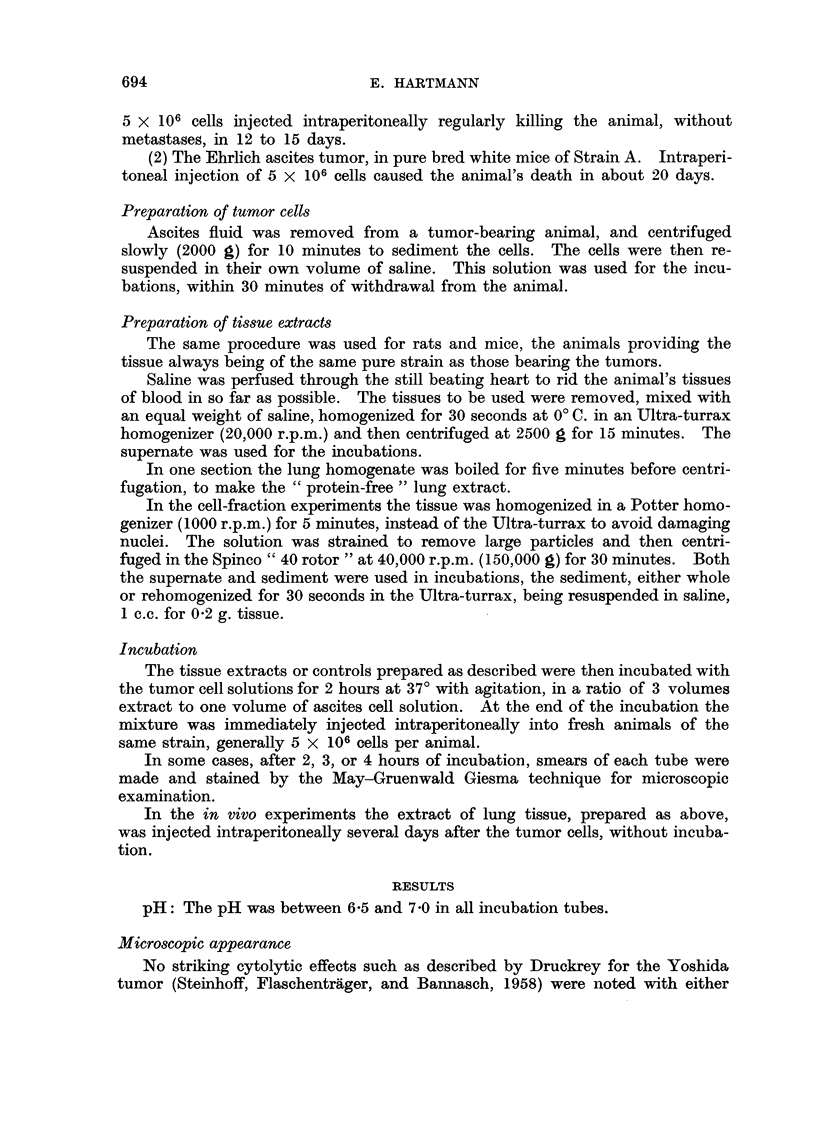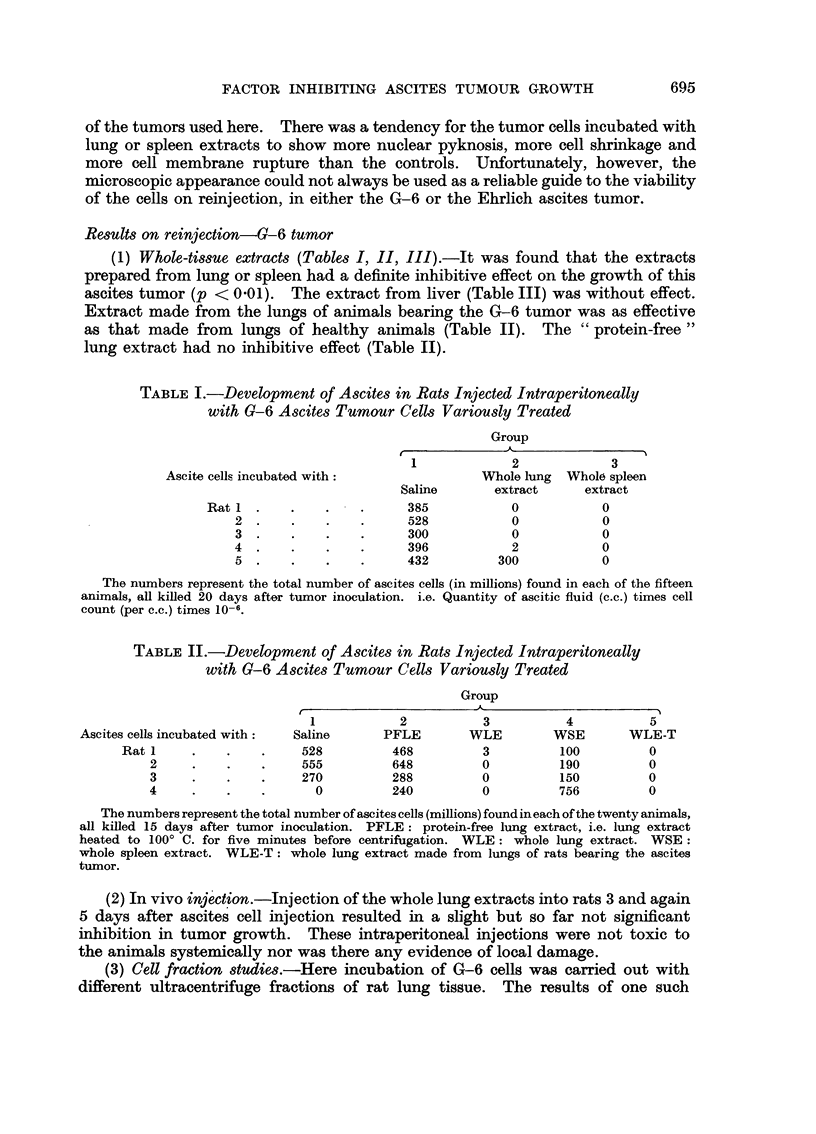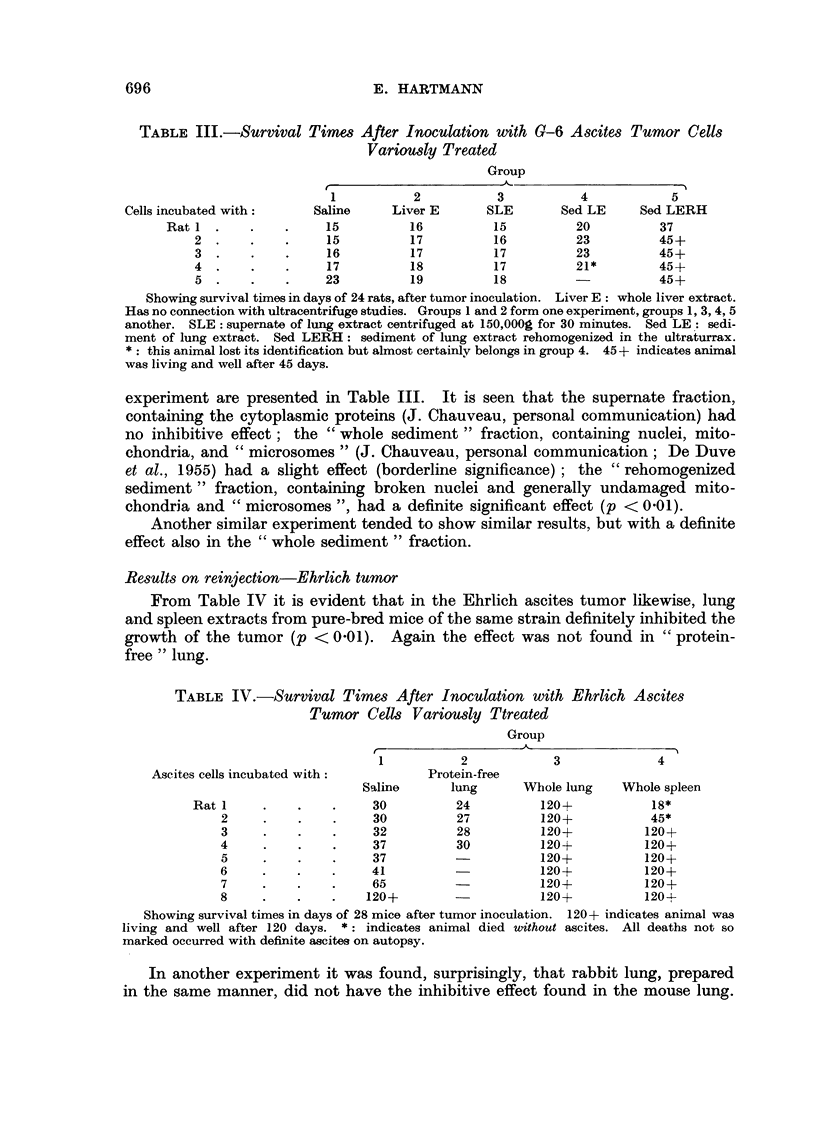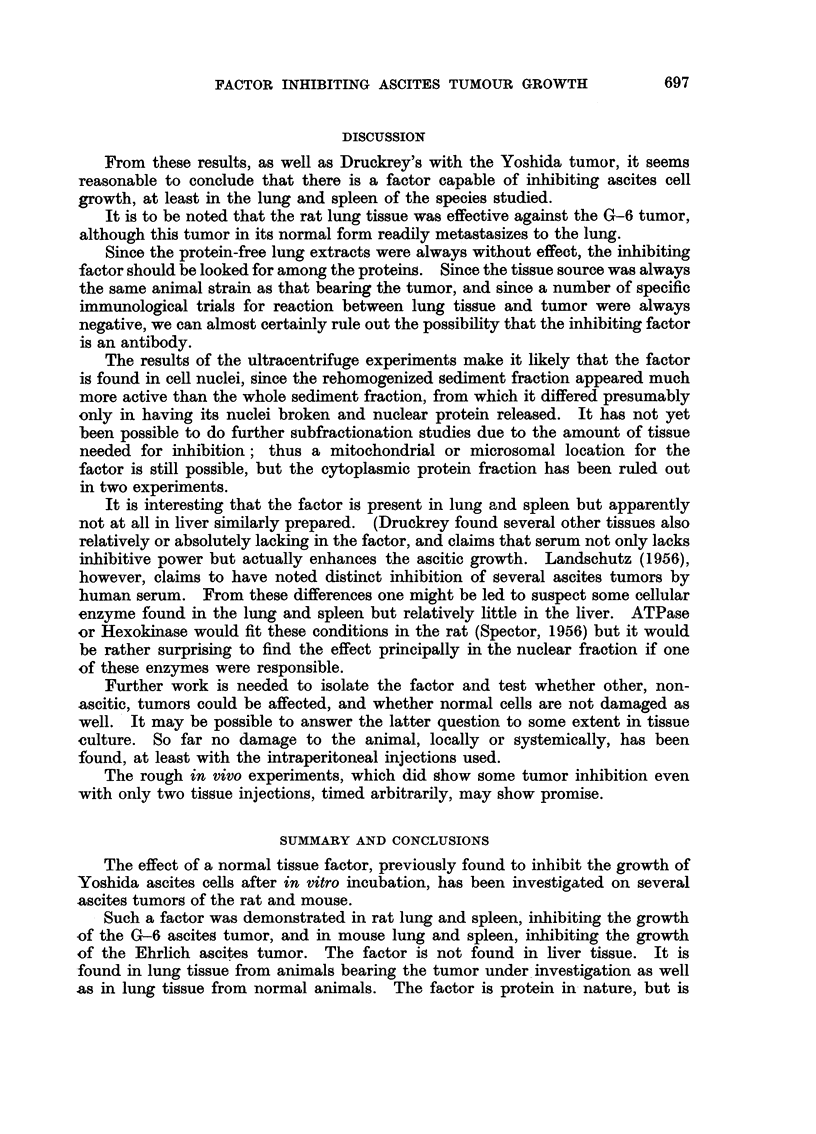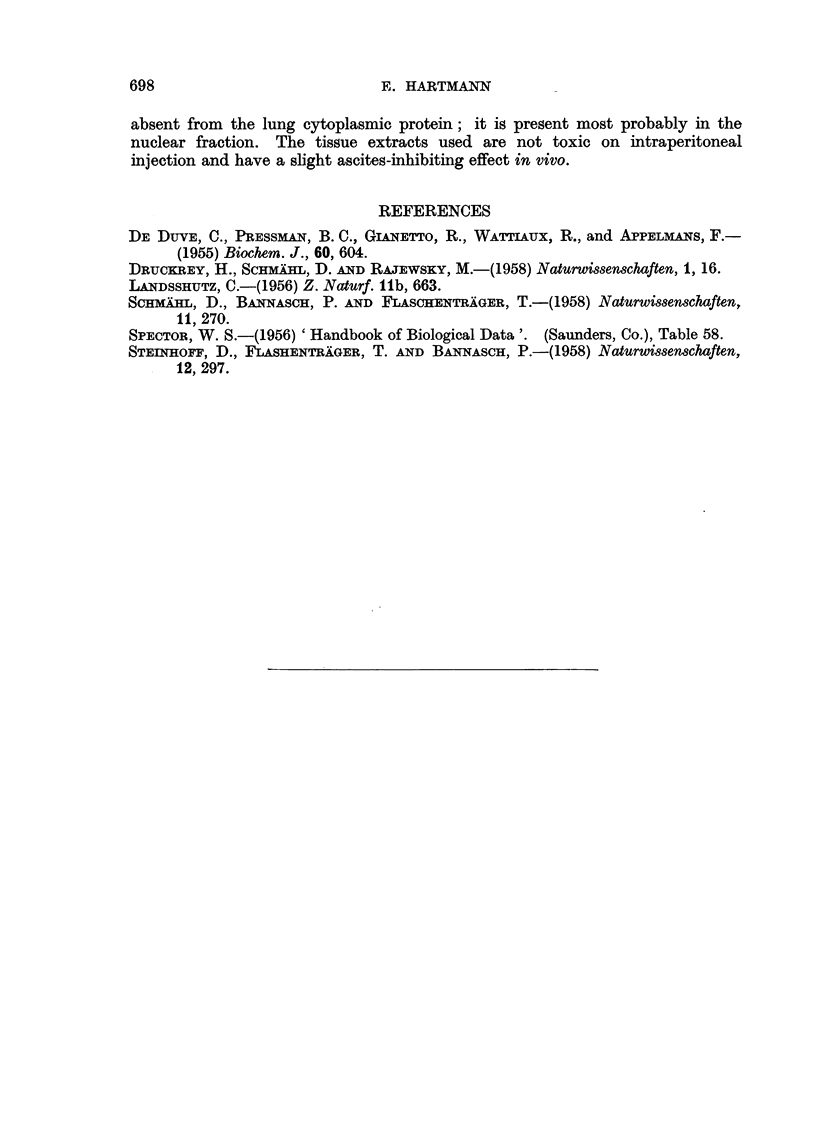# Investigations of a Factor Found in Certain Normal Tissues Inhibiting Ascites Tumor Growth in the Rat and Mouse

**DOI:** 10.1038/bjc.1959.77

**Published:** 1959-12

**Authors:** E. Hartmann


### INVESTIGATIONS OF A FACTOR FOUND IN CERTAIN NORMAL TISSUES INHIBITING ASCITES TUMOR GROWTH IN THE RAT AND MOUSE

E. HARTMANN

*From the Institut de Recherches sur le Cancer, Gustave Roussy, 16 bis, Avenue Vaillant-Couturier, Villejuif (Seine), France*

Received for publication September 3, 1959

It has long been known to pathologists that apparently normal cancer cells may circulate in the blood stream of cancer patients and eventually lodge in the lungs or other tissues where the great majority are inactivated and do not cause metastases. This may be merely a question of viability and the stroma-inducing ability of the cancer cells, but there is also a possibility that antibodies, or some local intrinsic tissue factor might be responsible. The last possibility has been under investigation by Druckey *et al.* for several years (Druckrey, Schmähl and Rajewsky, 1958 ; Steinhoff, Flaschenträger and Bannasch, 1958 ; Schmähl, Bannasch and Flaschenträger, 1958). Druckrey uses a simplified experimental system, consisting simply of incubating Yoshida ascites cells for several hours with various normal rat tissue homogenates, and then injecting the incubated cells intraperitoneally into rats of the same strain. Druckrey found that several tissue homogenates were responsible for a marked inhibition of tumor growth on reinjection (Druckrey, Schmähl and Rajewsky, 1958) and for microscopic damage to the tumor cells (Steinhoff, Flaschenträger and Bannasch, 1958). The effect was especially prominent with lung and spleen homogenates.

The experiments to be described were designed to elucidate further the nature of this phenomenon and the characteristics of the factor responsible. It was attempted :

(*a*) to verify Druckrey's results on other tumors and species;

(*b*) to eliminate by a series of checks and controls the possibility that the effect was due to physical or chemical properties of the tissue homogenates (pH, osmolarity, electrolyte composition) ;

(*c*) to see whether a tumor normally metastasizing to the lung would still be affected similarly by lung tissue ;

(*d*) to see whether tissue homogenates from tumor-bearing animals act in the same way as those from healthy animals ;

(*e*) to determine which cell fraction or fractions contain the responsible factor.

### MATERIALS AND METHODS

### *Species and tumors*

(1) The " G–6 " tumor in Wistar WAG pure bred white rats. This was originally a spontaneous mammary tumor of the rat ; in its solid transplantable form it metastasizes frequently to the lungs. The ascitic form used here had been passed in the Wistar rat for 30 generations and had attained a uniform pattern,

$5 \times 10^6$ cells injected intraperitoneally regularly killing the animal, without metastases, in 12 to 15 days.

(2) The Ehrlich ascites tumor, in pure bred white mice of Strain A. Intraperitoneal injection of $5 \times 10^6$ cells caused the animal's death in about 20 days.

*Preparation of tumor cells*

Ascites fluid was removed from a tumor-bearing animal, and centrifuged slowly (2000 **g**) for 10 minutes to sediment the cells. The cells were then resuspended in their own volume of saline. This solution was used for the incubations, within 30 minutes of withdrawal from the animal.

*Preparation of tissue extracts*

The same procedure was used for rats and mice, the animals providing the tissue always being of the same pure strain as those bearing the tumors.

Saline was perfused through the still beating heart to rid the animal's tissues of blood in so far as possible. The tissues to be used were removed, mixed with an equal weight of saline, homogenized for 30 seconds at 0° C. in an Ultra-turrax homogenizer (20,000 r.p.m.) and then centrifuged at 2500 **g** for 15 minutes. The supernate was used for the incubations.

In one section the lung homogenate was boiled for five minutes before centrifugation, to make the " protein-free " lung extract.

In the cell-fraction experiments the tissue was homogenized in a Potter homogenizer (1000 r.p.m.) for 5 minutes, instead of the Ultra-turrax to avoid damaging nuclei. The solution was strained to remove large particles and then centrifuged in the Spinco " 40 rotor " at 40,000 r.p.m. (150,000 **g**) for 30 minutes. Both the supernate and sediment were used in incubations, the sediment, either whole or rehomogenized for 30 seconds in the Ultra-turrax, being resuspended in saline, 1 c.c. for 0·2 g. tissue.

*Incubation*

The tissue extracts or controls prepared as described were then incubated with the tumor cell solutions for 2 hours at 37° with agitation, in a ratio of 3 volumes extract to one volume of ascites cell solution. At the end of the incubation the mixture was immediately injected intraperitoneally into fresh animals of the same strain, generally $5 \times 10^6$ cells per animal.

In some cases, after 2, 3, or 4 hours of incubation, smears of each tube were made and stained by the May–Gruenwald Giesma technique for microscopic examination.

In the *in vivo* experiments the extract of lung tissue, prepared as above, was injected intraperitoneally several days after the tumor cells, without incubation.

### RESULTS

pH: The pH was between 6·5 and 7·0 in all incubation tubes.

*Microscopic appearance*

No striking cytolytic effects such as described by Druckrey for the Yoshida tumor (Steinhoff, Flaschenträger, and Bannasch, 1958) were noted with either of the tumors used here. There was a tendency for the tumor cells incubated with lung or spleen extracts to show more nuclear pyknosis, more cell shrinkage and more cell membrane rupture than the controls. Unfortunately, however, the microscopic appearance could not always be used as a reliable guide to the viability of the cells on reinjection, in either the G–6 or the Ehrlich ascites tumor.

*Results on reinjection—G–6 tumor*

(1) *Whole-tissue extracts* (*Tables I, II, III*).—It was found that the extracts prepared from lung or spleen had a definite inhibitive effect on the growth of this ascites tumor ($p < 0{\cdot}01$). The extract from liver (Table III) was without effect. Extract made from the lungs of animals bearing the G–6 tumor was as effective as that made from lungs of healthy animals (Table II). The " protein-free " lung extract had no inhibitive effect (Table II).

TABLE I.—*Development of Ascites in Rats Injected Intraperitoneally with G–6 Ascites Tumour Cells Variously Treated*

| Ascite cells incubated with : | Group 1 Saline | Group 2 Whole lung extract | Group 3 Whole spleen extract |
|---|---|---|---|
| Rat 1 | 385 | 0 | 0 |
| 2 | 528 | 0 | 0 |
| 3 | 300 | 0 | 0 |
| 4 | 396 | 2 | 0 |
| 5 | 432 | 300 | 0 |

The numbers represent the total number of ascites cells (in millions) found in each of the fifteen animals, all killed 20 days after tumor inoculation. i.e. Quantity of ascitic fluid (c.c.) times cell count (per c.c.) times $10^{-6}$.

TABLE II.—*Development of Ascites in Rats Injected Intraperitoneally with G–6 Ascites Tumour Cells Variously Treated*

| Ascites cells incubated with : | Group 1 Saline | Group 2 PFLE | Group 3 WLE | Group 4 WSE | Group 5 WLE-T |
|---|---|---|---|---|---|
| Rat 1 | 528 | 468 | 3 | 100 | 0 |
| 2 | 555 | 648 | 0 | 190 | 0 |
| 3 | 270 | 288 | 0 | 150 | 0 |
| 4 | 0 | 240 | 0 | 756 | 0 |

The numbers represent the total number of ascites cells (millions) found in each of the twenty animals, all killed 15 days after tumor inoculation. PFLE : protein-free lung extract, i.e. lung extract heated to 100° C. for five minutes before centrifugation. WLE : whole lung extract. WSE : whole spleen extract. WLE-T : whole lung extract made from lungs of rats bearing the ascites tumor.

(2) In vivo *injection*.—Injection of the whole lung extracts into rats 3 and again 5 days after ascites cell injection resulted in a slight but so far not significant inhibition in tumor growth. These intraperitoneal injections were not toxic to the animals systemically nor was there any evidence of local damage.

(3) *Cell fraction studies*.—Here incubation of G–6 cells was carried out with different ultracentrifuge fractions of rat lung tissue. The results of one such

TABLE III.—*Survival Times After Inoculation with G–6 Ascites Tumor Cells Variously Treated*

| | Group | | | | |
|---|---|---|---|---|---|
| | 1 | 2 | 3 | 4 | 5 |
| Cells incubated with : | Saline | Liver E | SLE | Sed LE | Sed LERH |
| Rat 1 . . . | 15 | 16 | 15 | 20 | 37 |
| 2 . . . | 15 | 17 | 16 | 23 | 45+ |
| 3 . . . | 16 | 17 | 17 | 23 | 45+ |
| 4 . . . | 17 | 18 | 17 | 21* | 45+ |
| 5 . . . | 23 | 19 | 18 | — | 45+ |

Showing survival times in days of 24 rats, after tumor inoculation. Liver E : whole liver extract. Has no connection with ultracentrifuge studies. Groups 1 and 2 form one experiment, groups 1, 3, 4, 5 another. SLE : supernate of lung extract centrifuged at 150,000g for 30 minutes. Sed LE : sediment of lung extract. Sed LERH : sediment of lung extract rehomogenized in the ultraturrax. * : this animal lost its identification but almost certainly belongs in group 4. 45+ indicates animal was living and well after 45 days.

experiment are presented in Table III. It is seen that the supernate fraction, containing the cytoplasmic proteins (J. Chauveau, personal communication) had no inhibitive effect ; the " whole sediment " fraction, containing nuclei, mitochondria, and " microsomes " (J. Chauveau, personal communication ; De Duve *et al.*, 1955) had a slight effect (borderline significance) ; the " rehomogenized sediment " fraction, containing broken nuclei and generally undamaged mitochondria and " microsomes ", had a definite significant effect ($p < 0{\cdot}01$).

Another similar experiment tended to show similar results, but with a definite effect also in the " whole sediment " fraction.

*Results on reinjection—Ehrlich tumor*

From Table IV it is evident that in the Ehrlich ascites tumor likewise, lung and spleen extracts from pure-bred mice of the same strain definitely inhibited the growth of the tumor ($p < 0{\cdot}01$). Again the effect was not found in " protein-free " lung.

TABLE IV.—*Survival Times After Inoculation with Ehrlich Ascites Tumor Cells Variously Ttreated*

| | Group | | | |
|---|---|---|---|---|
| | 1 | 2 | 3 | 4 |
| Ascites cells incubated with : | Saline | Protein-free lung | Whole lung | Whole spleen |
| Rat 1 . . . | 30 | 24 | 120+ | 18* |
| 2 . . . | 30 | 27 | 120+ | 45* |
| 3 . . . | 32 | 28 | 120+ | 120+ |
| 4 . . . | 37 | 30 | 120+ | 120+ |
| 5 . . . | 37 | — | 120+ | 120+ |
| 6 . . . | 41 | — | 120+ | 120+ |
| 7 . . . | 65 | — | 120+ | 120+ |
| 8 . . . | 120+ | — | 120+ | 120+ |

Showing survival times in days of 28 mice after tumor inoculation. 120+ indicates animal was living and well after 120 days. * : indicates animal died *without* ascites. All deaths not so marked occurred with definite ascites on autopsy.

In another experiment it was found, surprisingly, that rabbit lung, prepared in the same manner, did not have the inhibitive effect found in the mouse lung.

### DISCUSSION

From these results, as well as Druckrey's with the Yoshida tumor, it seems reasonable to conclude that there is a factor capable of inhibiting ascites cell growth, at least in the lung and spleen of the species studied.

It is to be noted that the rat lung tissue was effective against the G–6 tumor, although this tumor in its normal form readily metastasizes to the lung.

Since the protein-free lung extracts were always without effect, the inhibiting factor should be looked for among the proteins. Since the tissue source was always the same animal strain as that bearing the tumor, and since a number of specific immunological trials for reaction between lung tissue and tumor were always negative, we can almost certainly rule out the possibility that the inhibiting factor is an antibody.

The results of the ultracentrifuge experiments make it likely that the factor is found in cell nuclei, since the rehomogenized sediment fraction appeared much more active than the whole sediment fraction, from which it differed presumably only in having its nuclei broken and nuclear protein released. It has not yet been possible to do further subfractionation studies due to the amount of tissue needed for inhibition ; thus a mitochondrial or microsomal location for the factor is still possible, but the cytoplasmic protein fraction has been ruled out in two experiments.

It is interesting that the factor is present in lung and spleen but apparently not at all in liver similarly prepared. (Druckrey found several other tissues also relatively or absolutely lacking in the factor, and claims that serum not only lacks inhibitive power but actually enhances the ascitic growth. Landschutz (1956), however, claims to have noted distinct inhibition of several ascites tumors by human serum. From these differences one might be led to suspect some cellular enzyme found in the lung and spleen but relatively little in the liver. ATPase or Hexokinase would fit these conditions in the rat (Spector, 1956) but it would be rather surprising to find the effect principally in the nuclear fraction if one of these enzymes were responsible.

Further work is needed to isolate the factor and test whether other, non-ascitic, tumors could be affected, and whether normal cells are not damaged as well. It may be possible to answer the latter question to some extent in tissue culture. So far no damage to the animal, locally or systemically, has been found, at least with the intraperitoneal injections used.

The rough *in vivo* experiments, which did show some tumor inhibition even with only two tissue injections, timed arbitrarily, may show promise.

### SUMMARY AND CONCLUSIONS

The effect of a normal tissue factor, previously found to inhibit the growth of Yoshida ascites cells after *in vitro* incubation, has been investigated on several ascites tumors of the rat and mouse.

Such a factor was demonstrated in rat lung and spleen, inhibiting the growth of the G–6 ascites tumor, and in mouse lung and spleen, inhibiting the growth of the Ehrlich ascites tumor. The factor is not found in liver tissue. It is found in lung tissue from animals bearing the tumor under investigation as well as in lung tissue from normal animals. The factor is protein in nature, but is absent from the lung cytoplasmic protein; it is present most probably in the nuclear fraction. The tissue extracts used are not toxic on intraperitoneal injection and have a slight ascites-inhibiting effect *in vivo*.

### REFERENCES

De Duve, C., Pressman, B. C., Gianetto, R., Wattiaux, R., and Appelmans, F.—(1955) *Biochem. J.*, **60**, 604.
Druckrey, H., Schmähl, D. and Rajewsky, M.—(1958) *Naturwissenschaften*, **1**, 16.
Landsshutz, C.—(1956) *Z. Naturf.* **11b**, 663.
Schmähl, D., Bannasch, P. and Flaschenträger, T.—(1958) *Naturwissenschaften*, **11**, 270.
Spector, W. S.—(1956) 'Handbook of Biological Data'. (Saunders, Co.), Table 58.
Steinhoff, D., Flashenträger, T. and Bannasch, P.—(1958) *Naturwissenschaften*, **12**, 297.